# Heart Failure and Atrial Fibrillation in Women: Pathophysiological Links, Clinical Challenges, and Therapeutic Perspectives

**DOI:** 10.3390/medicina62020261

**Published:** 2026-01-26

**Authors:** Luminiţa-Bianca Grosu, Camelia Cristina Diaconu, Laura Gabriela Gavril

**Affiliations:** 1 Faculty of Medicine, Carol Davila University of Medicine and Pharmacy Bucharest, 050474 Bucharest, Romania; luminita-bianca.grosu@drd.umfcd.ro; 2 Cardiology Department, Elias University Emergency Hospital, 011461 Bucharest, Romania; 3 Internal Medicine Department, Clinical Emergency Hospital of Bucharest, 105402 Bucharest, Romania; 4 Academy of Romanian Scientists, 050085 Bucharest, Romania; 5 Faculty of Medicine, Grigore T. Popa University of Medicine and Pharmacy, 700115 Iasi, Romania; cotirlet_laura@yahoo.com; 6 Department of Anaesthesiology and Intensive Care, Regional Institute of Oncology, 700483 Iasi, Romania

**Keywords:** women, heart failure, atrial fibrillation, ejection fraction, sex differences

## Abstract

*Background and Objectives*: The prevalence of heart failure and atrial fibrillation is increasing because of population aging. There are important sex-related differences in the epidemiology, pathophysiology, treatment, and prognosis of patients with both heart failure and atrial fibrillation. While the overall lifetime risk of both diseases is similar between women and men, women tend to be older when diagnosed and to have more comorbidities. *Materials and Methods*: A narrative review was conducted by analyzing studies published across databases such as PubMed, SCOPUS, Web of Science, and Google Scholar. The review focused on research about sex-related differences in patients with heart failure and atrial fibrillation, emphasizing the peculiarities in women regarding drug treatment and prognosis after cardiac device implantation. *Results*: Current evidence highlights the sex-related differences in patients with both heart failure and atrial fibrillation regarding pathophysiology, clinical manifestations, and echocardiographic findings. There are also data regarding possible sex-related differences in mortality and therapy, as women tend to have longer hospital stays, but there are fewer reevaluations after discharge. *Conclusions*: Women with both atrial fibrillation and heart failure are at increased risk of stroke and other adverse outcomes that negatively affect their quality of life. Females with atrial fibrillation and heart failure tend to be treated less with rhythm control strategies and ablation, which may have a great impact on symptom burden in women compared to men.

## 1. Introduction

Heart failure and atrial fibrillation are common cardiovascular diseases [1], with increasing prevalence and consequences on quality of life, morbidity, and mortality. Smoking, hypertension, obesity, diabetes, and coronary heart disease are risk factors playing central roles in the development of these two conditions [2,3]. Moreover, a bidirectional connection exists between heart failure and atrial fibrillation, as one-third of patients with atrial fibrillation develop heart failure, while more than half of patients with heart failure, regardless of left ventricle ejection fraction, develop atrial fibrillation [2,4].

In terms of global disease burden, in 2019, there was almost a 50% increase in the number of patients with atrial fibrillation and associated heart failure compared with 1990. This rise may be related to positive changes in healthcare systems, easier access to healthcare professionals, earlier diagnosis, and population aging [5].

Sex-related differences have been reported in epidemiology, access to the healthcare system, impact of other comorbidities, and adherence to treatment. There is concern that women are still underrepresented in cardiovascular trials, which may result in uncertainty and in the extrapolation of study outcomes from males to females [6,7]. Two groups are extremely rarely included in trials: pregnant women and those of childbearing age [8,9].

Sex-related differences exist from clinical examination to the selection of the most appropriate therapy. Women with atrial fibrillation are older, more symptomatic, and sometimes have a lower quality of life and access to the newest rhythm control strategies. Furthermore, women have more frequent atrial fibrillation-associated complications (heart failure, stroke) and a higher risk of all-cause mortality [10,11,12]. In addition, some studies emphasize differences in drug doses, as women are treated with lower doses of angiotensin-converting enzyme inhibitors, angiotensin receptor blockers, and β-blockers, while men benefit from the full target doses [13,14]. Therefore, the underrepresentation of women in clinical trials can lead to potential harm and more side effects at the same doses [14,15].

## 2. Materials and Methods

To conduct this comprehensive narrative review on the research about sex-related differences in patients with heart failure and atrial fibrillation, with an emphasis on the peculiarities regarding drug treatment and prognosis after cardiac device implantation in women, a systematic strategy was employed to identify relevant literature across multiple databases.

The following databases were utilized due to their extensive coverage of biomedical and technological research: Pubmed, SCOPUS, Web of Science, and Google Scholar.

- Search Strategy

The literature search was carried out using a combination of keywords and Boolean operators to identify a broad range of studies related to women with heart failure and atrial fibrillation. The search queries were tailored to each database’s specific requirements, as emphasized below:

The search strategy incorporated a combination of keywords:

(1) Women with heart failure: “estrogen”, “fatigue”, “ejection fraction”, “dyspnea”.
(2) Women with atrial fibrillation: “palpitations”, “anticoagulants”, “ablation”, “heart fibrosis”, “rhythm control”.

Boolean operators, such as “AND” and “OR”, were used to combine these keywords effectively, allowing for a comprehensive search that included all the relevant literature.

- Inclusion and Exclusion Criteria

The selection of studies for inclusion in this review was guided by specific inclusion and exclusion criteria to ensure relevance and quality. We included articles published in English that were reviews, clinical studies, or original research articles focusing on sex-related differences in pathophysiology, clinical aspects, echocardiography, and treatment in patients with heart failure and atrial fibrillation.

Exclusion criteria included non-English articles, articles without accessible full texts, studies not directly related to women with heart failure and atrial fibrillation, opinion pieces, conference abstracts without full papers, and editorials.

Reference lists of included articles were examined to identify additional relevant studies. Citation databases were used to find newer articles citing the included studies.

- Rationale for Design and Data Synthesis

This review employs a narrative rather than systematic approach due to the heterogeneity of current literature and the necessity of sex-differentiating clinical aspects and therapies in patients with heart failure and atrial fibrillation—diseases which frequently coexist, affecting every aspect of life. The existing research spans diverse specialties, including cardiology, endocrinology, and public health, utilizing varied methodologies and study designs, which complicates standardization under systematic review protocols. Additionally, many studies are descriptive, lacking the structured outcomes needed for meta-analytical synthesis. Consequently, a narrative synthesis was chosen as the most appropriate method to include findings and challenges and address future direction. This narrative review attempts to present a comprehensive overview of the current evidence and offer possible directions for better understanding sex-related differences.

## 3. Epidemiology of Heart Failure and Atrial Fibrillation

Heart failure and atrial fibrillation often coexist, resulting in poorer cardiovascular outcomes. Epidemiological studies have shown that in patients with preexisting atrial fibrillation, the development of heart failure led to rates of mortality three times higher in both men and women [16]. In patients with preexisting heart failure, the development of atrial fibrillation led to a significant increase in mortality in women compared to men [3,12].

Important risk factors for developing atrial fibrillation and heart failure in women are smoking, obesity, hypertension, and diabetes [12]. The Women’s Health Study has shown that women with newly diagnosed atrial fibrillation had a higher risk of developing heart failure. Moreover, when women with preexisting atrial fibrillation developed heart failure, a major increase in all-cause mortality was observed [17]. The modification of the risk factors mentioned above may diminish the development of heart failure and its consequences in women with atrial fibrillation [12]. When comparing the lifetime risk of heart failure phenotypes by sex, epidemiological studies show that heart failure with preserved ejection fraction (HFpEF) is more prevalent in women, while men tend to develop heart failure with reduced ejection fraction (HFrEF), regardless of age [18]. The risk of developing both atrial fibrillation and heart failure is higher in women than in men [12]. Moreover, women with preexisting atrial fibrillation have a higher incidence of HFpEF compared to men [19,20].

## 4. Pathophysiology of Heart Failure and Atrial Fibrillation

The mechanisms of atrial fibrillation and heart failure are highly complex, including structural, genetic, and physiological heart changes. When considering sex differences, hormonal influences play crucial roles in the incidence and pathophysiology of heart failure and atrial fibrillation.

Emerging research suggests that sex-related differences in heart failure and atrial fibrillation are partly determined by epigenetic mechanisms, such as microRNA expression, histone changes, and DNA methylation. These mechanisms influence gene expression involved in myocardial fibrosis, hypertrophy, and electrical remodeling [21].

- Sex differences in heart failure pathophysiology

The different prevalence and incidence of heart failure phenotypes in women versus men are determined by mechanisms such as sex-specific gene expression, immune responses, gender differences in cardiac structure, impact of sex steroids on cardiac cells, endothelial cells, and vascular smooth muscle cells, as well as heart failure risk factors and patient comorbidities [21].

Some studies observed that women develop HFpEF more often than men, who develop HFrEF more frequently. These predispositions can be explained by sex-related differences in heart structure and function: women have cardiac index similar to that of men, but with smaller indexed left ventricle (LV) volumes and higher LV stiffness [21]. Moreover, when considering afterload stress, women often maintain a LV preserved ejection fraction for a long time and promote LV hypertrophy and diastolic dysfunction (due to smaller aortic root and aortic arch, which leads to higher pulse pressure and impaired coronary flow in women), while men develop eccentric remodeling (cardiomyocytes in males have higher density of β1-adrenergic receptors, and their prolonged stimulation determines increased collagen release with LV dilation and eccentric remodeling) [21,22].

**Figure 1:**
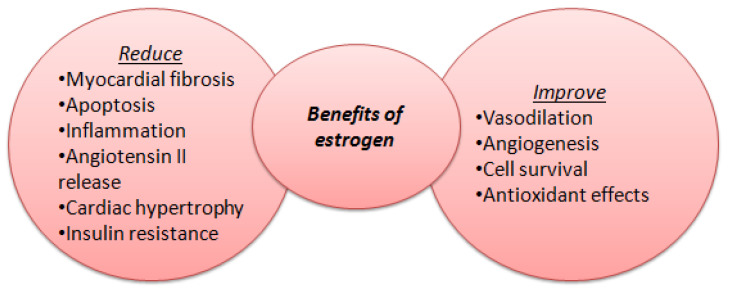
Benefits of estrogen levels on cardiac function.

Besides differences in structure and function, recent studies emphasized the major role of systemic inflammation and its consequences (endothelial dysfunction, microvascular dysfunction, ischemia, fibrosis) in developing HFpEF [23]. Endothelial dysfunction may be triggered by menopause, when antioxidant and anti-inflammatory roles of oestradiol (E2) decrease [24]. In premenopausal women estrogens are synthesized mainly in the ovaries, corpus luteum, and placenta and have a crucial protective role against cardiovascular disease. Estrogens have cardioprotective effects through genomic and non-genomic pathways, mediated by estrogen receptors ERα, ERβ, and the G protein-coupled estrogen receptor (GPER). Activation of these receptors modulates oxidative stress, calcium intake, mitochondrial function, and inflammatory processes, influencing myocardial contractility and electrical stability (Figure 1). Estrogens diminish ventricular remodeling and myocardial fibrosis. After menopause, the estrogen levels decrease, explaining the increased susceptibility of females to heart failure with preserved ejection fraction and atrial fibrillation at older ages [24]. Women tend to have more proinflammatory myocardium genes, which contribute to a higher prevalence of autoimmune responses, than men, and consequently a higher risk of autoimmune diseases [25,26].

In addition, biological tests in women show higher circulating levels of natriuretic peptides, cardiovascular biomarkers with stronger predictive value [27,28].

- Sex differences in the pathophysiology of atrial fibrillation

Hormones have significant effects on atrial tissue, contributing to cardiac remodeling and arrhythmogenesis, especially in postmenopausal women [29]. Atrial fibrillation is triggered by hormones such as catecholamines, aldosterone, angiotensin II, thyroid hormones, natriuretic peptides, cortisol, and sex hormones (estrogen, progesterone) through mechanisms like ion channel dysfunction, activation of inflammatory pathways, autonomic nervous system activation, and atrial fibrosis [25,30].

Sex hormones have important effects on cardiac tissue, as females develop different patterns of atrial remodeling compared to men. After menopause, the estrogen levels decrease, and consequently, sympathetic activity is enhanced, affecting heart rate and conduction properties and promoting the risk of atrial fibrillation [31,32]. Besides estrogen, another crucial sex hormone involved in modulating atrial fibrillation occurrence is progesterone; together, they influence the autonomic nervous system, which mediates atrial fibrillation development [25]. Studies have found that during the follicular phase, the parasympathetic system is more active, while during the luteal phase, sympathetic system activity is increased, predisposing women to arrhythmias [33,34]. Other factors which influence the autonomic nervous system are sex-related differences in cardiac structure and function, as females have reduced heart dimensions and different atrial organization [11,25]. Autonomic nervous system responses and remodeling are sex-differentiated, with women experiencing more emphatic autonomic reactions in response to atrial fibrillation, which can determine more disease-related complications [25,35].

**Figure 2:**
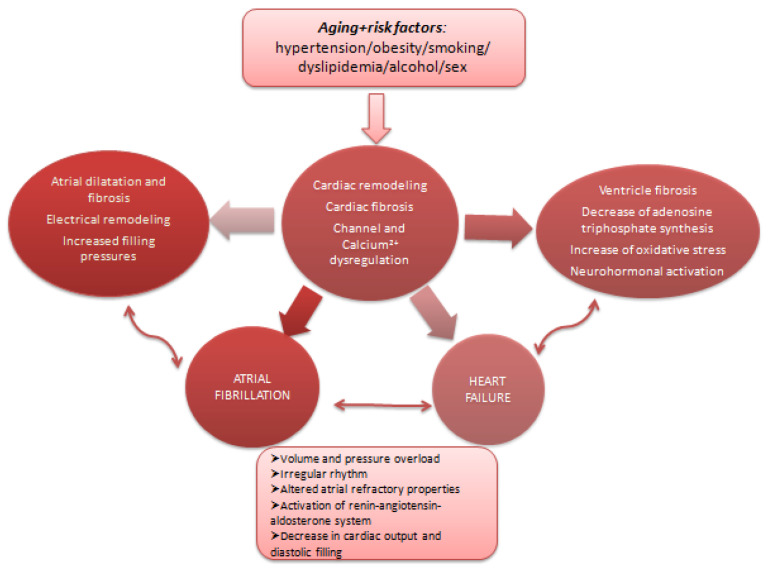
Common pathophysiological pathways for heart failure and atrial fibrillation.

In addition, sex hormones influence structural changes (different patterns of atrial fibrosis, atrial enlargement) associated with atrial fibrillation, estrogen having a protective role before menopause against cardiac fibrosis and in reducing atrial fibrillation evolvement [36] (Figure 2). Estrogen signaling appears to modulate fibroblast activation and collagen deposition through transforming growth factor β (TGF-β)-dependent pathways. Enhanced myocardial stiffness and diastolic dysfunction may predispose women to heart failure with preserved or mildly reduced ejection fraction, while atrial fibrosis contributes to atrial fibrillation initiation and maintenance [36]. Imaging studies have shown that women have smaller atrial volumes, which may influence conduction times and predisposition to arrhythmias, whilst men have larger atrial volumes and a higher rate of success in cardioversion for atrial fibrillation, suggesting sex-related differences in atrial electrophysiology (women with atrial fibrillation are characterized by lower atrial voltage—surrogates for atrial fibrosis, slower conduction velocity, and more complex fractionated potentials) [37,38].

## 5. Clinical Presentation and Diagnosis

The most frequent symptoms caused by atrial fibrillation are palpitations, dyspnea, chest pain, and dizziness. Women are usually more symptomatic than men, presenting with atypical symptoms (fatigue, weakness) with longer duration than in men, whilst males tend to have more typical symptoms (palpitations, shortness of breath). The presence of atypical symptoms sometimes delays the diagnosis of atrial fibrillation and, consequently, the early initiation of treatment, with severe outcomes in females [13].

Similar to atrial fibrillation, women with heart failure tend to be more symptomatic, with signs of congestion and a worse quality of life compared to men. The rate of hospitalization in patients with heart failure is similar in both sexes, but with a higher risk of cardiovascular death in men [13].

There are many tools used to diagnose and monitor the impact of heart failure and atrial fibrillation on disease progression. The most-used prognosis biomarker in patients with heart failure is N-terminal pro-B-type natriuretic peptide (NT-proBNP). Increased levels of NT-proBNP seem to correlate with age and are more prevalent in women than in men. This can be explained by the effects of sex hormones, as some hypotheses suggest that testosterone controls neprilysin activity and reduces NT-proBNP levels [28].

Regarding transthoracic echocardiography, studies have shown that in women with HFpEF, ejection fraction (EF) is higher than in males with HFpEF, with lower LV end-diastolic and end-systolic volume index, lower LV mass indexed to body surface area, less LV dilation, but similar LV hypertrophy [39]. Left atrial volume index and pulmonary artery systolic pressure were observed to be similar in females and males. Diastolic dysfunction was shown to be similar in both men and women with HFpEF (high mean mitral inflow velocity to diastolic mitral annular velocity at early filling E/e’ ratio, high E/A ratio, E-wave velocity, A-wave velocity, tricuspid regurgitation velocity), being frequently diagnosed in patients with heart failure and atrial fibrillation. However, some studies have shown that women have a higher E/e’ ratio, with increased LV filling pressure and higher mitral inflow velocity (A wave velocity), due to greater LV stiffness, especially in HFpEF [19,40].

## 6. Therapeutic Management of Patients with Heart Failure and Atrial Fibrillation

### 6.1. Pharmacological Therapy

Current guidelines do not have different recommendations for men and women, even though increasing evidence emphasizes sex-related differences in drug efficacy and safety. Women tend to need reduced doses of drugs for heart failure and atrial fibrillation because of differences in renal and hepatic metabolism and clearance, body fat distribution, body weight, height, and hormonal status. Beyond their hemodynamic effects, several heart failure therapies exert pleiotropic actions that can be particularly relevant in females. A series of studies shows that women benefit more from a submaximal drug dose, while recommended target doses (angiotensin-converting enzyme inhibitors (ACEIs), angiotensin II receptor blockers (ARBs), beta-blockers) are more effective in men [21,41]. Women experience more adverse reactions to certain drugs and a negative survival impact [21,41,42].

Current heart failure treatment includes ACEIs or ARBs, beta-blockers, mineralocorticoid receptor antagonists or angiotensin receptor-neprilysin inhibitor (ARNI), and sodium-glucose transport protein 2 inhibitors (SGLT2i) [21].

Trials such as The Acute Infarction Ramipril Efficacy (AIRE) study and the Heart Outcomes Prevention Evaluation (HOPE) study indicated that ACEIs confer the same benefit in males and females, with an almost identical reduction in mortality [21]. Later, The BIOlogy Study to Tailored Treatment in Chronic Heart Failure (BIOSTAT-HF) showed that in patients diagnosed with HFrEF, the decrease in cardiovascular events and mortality was similar between men and women, even when women received a smaller dose of ACEI, without up-titrating to guideline-recommended doses [41,43].

Among HFrEF patients, women have a higher survival benefit after introducing ARBs due to the lower rate of adverse effects, while among HFpEF patients, there are no significant sex-related differences [13,21,44].

Beta-blockers are associated with essential survival benefits in both women and men with heart failure, but some studies show greater pharmacodynamic effects of smaller doses of beta-blockers in women, as they have a more pronounced parasympathetic system activity and reduced activity of the liver cytochrome CYP2D6 [43]. Contraceptive pills interact with metoprolol metabolism and increase its plasma levels [13,45].

Angiotensin-converting enzyme inhibitors and angiotensin receptor blockers reduce myocardial fibrosis and inflammation, while beta-blockers influence autonomic regulation and electrical remodeling [21].

Regarding the mineralocorticoid receptor antagonists, the Treatment of Preserved Cardiac Function Heart Failure with an Aldosterone Antagonist (TOPCAT) trial demonstrated a decreased mortality in women with HFpEF or heart failure with mid-range ejection fraction (HFmrEF) compared to men treated with spironolactone, while efficacy results were similar in HFrEF patients of both sexes [46].

The Prospective Comparison of ARNI with ACEI to Determine Impact of Global Mortality and Morbidity in Heart Failure (PARADIGM-HF) trial on HFrEF patients showed a similar decrease in mortality in both sexes [47], while the Prospective Comparison of ARNI with ARB Global Outcomes in HFpEF (PARAGON-HF) trial, which included patients with HFpEF and HFmrEF, demonstrated an important reduction in hospitalizations for heart failure and cardiovascular death rate, especially in women [44]. Moreover, the Prospective Study of Biomarkers, Symptom Improvement, and Ventricular Remodeling During Sacubitril/Valsartan Therapy for Heart Failure (PROVE-HF) trial showed significant reductions in NT-proBNP, improvement in health status, and reverse cardiac remodeling in women with HFrEF [9]. In addition, women with HFpEF tend to be more responsive to ARNI than men due to lower NT-proBNP levels after menopause, increased neprilysin activity because of greater visceral adipose tissue, differences in regulation of nitric oxide synthases, and microvascular inflammation [48].

Studies demonstrate that in HFrEF patients, SGLT2i have greater efficacy in endpoints such as worsening heart failure or cardiovascular death in females, whilst in HFpEF patients, SGLT2i have similar outcomes in both sexes [41]. Even though SGLT2i have more side effects in women (urinary tract and genital mycotic infections), they tend to show almost the same efficacy and safety in both women and men with diabetes mellitus [42,49]. Moreover, emerging studies suggest potential sex-specific effects of SGLT2i on myocardial metabolism, oxidative stress, and endothelial function, thereby reducing cardiovascular outcomes in the cardio-renal-metabolic syndrome and, more importantly, reducing the relative risk of sudden cardiac death across different populations [49].

### 6.2. Rate Versus Rhythm Control Strategy in Patients with Atrial Fibrillation and Heart Failure

The Atrial Fibrillation Follow-up Investigation of Rhythm Management (AFFIRM) and The Rate Control Versus Electrical Cardioversion (RACE) trials observed no differences in cardiovascular mortality between rate versus rhythm control strategies in patients with HFrEF in the New York Heart Association functional classes II–IV [12]. According to the AFFIRM trial, women have a higher risk of stroke compared to men, even though the risk of death associated with rate versus rhythm control strategies is similar in both sexes [12]. According to the RACE trial, females treated with a rhythm control strategy had an increased incidence of thromboembolic events, cardiovascular mortality, and side effects of antiarrhythmic drugs compared to women treated with rate control strategy [12].

Women have been proven to be more susceptible to longer corrected QT intervals and torsades de pointes than men when treated with amiodarone, disopyramide, quinidine, sotalol, terfenadine, ibutilide, and erythromycin [50]. In addition, studies showed that women are more likely to receive digoxin therapy and fewer beta-blockers. A post hoc analysis showed that women with heart failure treated with digoxin had a higher risk of mortality compared to those treated with placebo, and this effect was not observed in males [12,13,21,51].

Catheter ablation for rate control may be an excellent alternative to drug therapy for improving quality of life, LVEF, and functional capacity; however, studies including more males have shown that women rarely undergo ablation for atrial fibrillation because of older age at diagnosis and higher risk of complications [52,53,54].

Recurrence of atrial fibrillation after catheter ablation is more frequent in females because of lower atrial voltage, slower conduction velocity, and greater degrees of atrial fibrosis [53].

### 6.3. Anticoagulation and Stroke Risk

Studies have shown that women tend to have more arrhythmia-related symptoms and a greater risk of stroke, despite men having a higher lifetime risk of atrial fibrillation, a difference correlated with cardiac structure and electrophysiology (increased atrial fibrosis and greater risk of progression to atrial fibrillation because of hormonal fluctuations in women, larger atrial dimensions in males) [55].

The 2024 European Society of Cardiology guidelines for the management of atrial fibrillation highlight that female sex represents an age-dependent stroke risk modifier rather than an independent biological risk factor when considering the CHA_2_DS_2_-VA score, as studies have shown that the highest stroke risk in females is in older populations (>65 years) [56]. Despite the known greater risk of stroke, women tend to receive less anticoagulant treatment because of higher hemorrhage risk (especially of vitamin K antagonists) or sex-related differences in cardiovascular care. The exclusion of sex from the CHA_2_DS_2_-VA score represents a step forward to a sex-specific therapeutic strategy for stroke prevention [57,58].

### 6.4. Device Therapy

Devices for heart failure include implantable cardioverter defibrillators (ICD), cardiac resynchronization (CRT), and CRT with defibrillators (CRT-D) [21].

The rates of ICD implantation are similar in both sexes, but with higher risks of complications in women (pneumothorax, hemorrhage, local infection, lead dislodgement) [59,60].

Studies have found that women benefit more from CRT and CRT-D implantation with respect to outcomes such as quality of life, rate of hospitalization, reverse cardiac remodeling, and cardiovascular survival [61,62]. The positive effects can be explained by the females’ reduced rate of ischemic etiology of heart failure, reduced scar tissue, and body index (including heart dimensions) [63]. No sex-specific recommendations for device therapy are indicated in the guidelines, as women might need lower cut-off values of QRS duration for device implantation [21,40,61].

## 7. Psychosocial and Gender-Related Factors

*Traditional risk factors.* One important risk factor for HF is obesity (body mass index ≥ 27.5 kg/m^2^ and waist circumference > 90 cm in females or >100 cm in males), which is particularly associated with HFpEF and is more frequent in women [64]. Some studies correlate the higher incidence of HFpEF with the dimension of visceral tissue area in women, whilst other studies explain female susceptibility to obesity as being related to low estrogen levels at menopause, which reduce the protective role of microRNA and correlate with an increase in insulin resistance that favors systemic inflammation and endothelial dysfunction. Another significant risk factor is hypertension, which is also more prevalent in females [48,65]. Women and men have different remodeling patterns, with females developing LV concentric hypertrophy because of hypertension. In addition, women have more significant arterial stiffness than men, which leads to increased systolic load and cardiac afterload, favoring diastolic dysfunction of the LV [66]. Another risk factor that contributes to diastolic dysfunction is diabetes mellitus, which affects small myocardial blood vessels, maintains systemic inflammation and endothelial dysfunction, and activates the release of reactive oxygen species. These processes are similar in males and females, but they tend to be initiated earlier in females [21,67].

*Socioeconomic factors.* Women with lower access to education have a higher susceptibility to high blood pressure, myocardial infarction, obesity, dyslipidemia, heart failure, and alcohol consumption compared to men with the same educational possibilities. Regarding smoking, both sexes tend to have similar habits in the case of low education. Moreover, marital status, diet, and environmental activities may be different in females versus males and play a major role in cardiovascular risk [65,68]. People with less access to the education system and who are socially disadvantaged are more prone to having an insufficient access to healthcare providers, financial instability, and unhealthy behaviors (poor nutrition, alcohol consumption, smoking), which are more pronounced in females [69,70].

*Mental health.* Studies show that depression increases the risk of cardiovascular disease in both sexes, but the association is stronger in women, especially after age 50. One explanation is that women tend to experience more anxiety and depression during specific periods, such as pregnancy and menopause. Another explanation is that women are more frequently diagnosed with depression and have more exposure to traditional risk factors. This association, along with differential treatment and healthcare in females, can contribute to the development of cardiovascular diseases. However, women receive less treatment for their mental health issues [21,71].

*Caregiver role.* Historically, women identify as caregivers for the family and society; on the one hand, this gives a meaning and a sense of connection to family members and community, giving women the chance to reach a balance between their multiple roles. On the other hand, the lack of self-care leads to impairment of the female’s caregiving role, affecting multiple areas of interest. It is necessary to acquire a balance between caregiving responsibilities and encouraging strategies and activities for better cardiovascular health, as women represent a higher proportion of the cardiovascular population than men [72,73].

## 8. Outcome and Prognosis

Recent studies show that the rates of re-hospitalization, morbidity, and mortality are higher in males. This susceptibility can be explained by the fact that male patients have a higher incidence of HFrEF, which has a worse long-term prognosis. Moreover, some trials observed that, when compared to men with HFpEF, female patients with HFpEF still have a lower mortality; this association was also demonstrated when comparing females and males with HFrEF, women diagnosed with HFrEF having fewer hospitalizations and longer survival rates. However, even though women live longer, their quality of life is poorer because of a greater number of comorbidities and complications, more heart failure symptoms, and greater psychological disability, with frequent episodes of anxiety and depression [12,21].

Studies have shown that the health-related quality of life (HRQL) is worse in females due to demographic factors, symptom burden, and low functional status [73]. The impact of heart failure diagnosis on psychological status is greater in females, as they have more anxiety and depression manifestations than men. Consequently, HRQL scores are lower in females, regardless of age or stage of disease, because of lower social support and, sometimes, differential medical and device therapies (longer hospital stays, lower rates of ICD, or CRT implantation in females) [73,74].

Regarding atrial fibrillation, females have a worse HRQL compared to men and report more symptoms. Moreover, when comparing administration of antiarrhythmic drugs with electrical cardioversion, studies observed an improvement in HRQL and the severity and frequency of symptoms in both sexes, but it was still worse in women after drug therapy [75]. After ablation for atrial fibrillation, some studies showed a significant amelioration of HRQL and symptom frequency in both sexes, with improvement in physical scores in women and in mental scores in men [76,77].

Some studies noted that women are more sensitive to symptoms and have a different perception of disease and way of responding to it than men, suggesting that females’ quality of life may be influenced more due to these factors and less to atrial fibrillation per se. In addition, anxiety and depression, which are more frequent among females, lead to a poorer HRQL [76,78,79].

## 9. Gaps in Research and Future Directions

There are substantial gaps in our knowledge of sex-related differences regarding incidence, prevalence, risk factors, prognosis, and especially medical therapies of heart failure and atrial fibrillation in women and men.

There is a lack of sex-related mortality differences due to in-hospital therapies, as women tend to have longer hospital stays but with reduced numbers of LVEF reevaluations or CRT/ICD implantations [74]. Sex-specific indications for CRT/ICD implantation are necessary, as females are responsive to CRT therapy at shorter QRS durations than men [40], emphasizing the need for lower cut-off values for QRS duration in women. Future studies should aim to define sex-specific criteria for CRT and ICD implantation, leading to personalized therapeutic thresholds.

Moreover, there is a lack of studies regarding patients with HFmrEF, especially in women [80]. Future research should prioritize prospective, sex-stratified analyses, as females tend to remain underrepresented in most clinical trials involving atrial fibrillation and heart failure (particularly relevant for patients with HFmrEF, as this population is insufficiently studied).

In addition, females have a greater risk of side effects of cardiovascular medication due to polymorphic genetic mechanism responses to ACEI, beta-blockers, calcium-channel blockers interfering with sex hormone actions, menstrual cycles, comorbidities, and other drugs [21,81,82,83]. There is a need for special studies evaluating sex-related differences in pharmacokinetics, pharmacodynamics, and the adverse effects of cardiovascular drugs. Genetic polymorphisms, comorbidities, and hormonal impact can contribute to a higher incidence of drug-related side effects in women, highlighting the importance of personalized treatment strategies.

Even though the pathophysiology of heart failure and atrial fibrillation has been studied for years, our knowledge of sex-related differences regarding cardiac anatomy and electrical pathways and mechanisms combined with hormonal differences remains insufficient.

Finally, future studies should place greater emphasis on HRQL and psychosocial outcomes to determine the factors that affect these indicators most in order to guide social support along with medical therapies for females with heart failure and atrial fibrillation.

## 10. Conclusions

Even though atrial fibrillation occurs more frequently in men, females with atrial fibrillation tend to have more pronounced symptoms, worse LV systolic and diastolic profiles, and a higher risk of adverse cardiovascular outcomes.

Although heart failure represents one of the leading causes of morbidity and mortality worldwide, females are still underrepresented in clinical trials, deepening the gap in optimizing heart failure care addressed to them.

Sex-related differences affect every characteristic of heart failure and atrial fibrillation, taken separately or together, from epidemiology, clinical manifestations, and pathophysiology to drug and device therapies and quality of life. There are still significant gaps in optimal drug and interventional treatment for heart failure and atrial fibrillation.

## Data Availability

No new data were created or analyzed in this study. Data sharing is not applicable to this article.

## Abbreviations

The following abbreviations are used in this manuscript:

ACEIs	Angiotensin-Converting Enzyme Inhibitors
ARBs	Angiotensin II Receptor Blockers
ARNI	Angiotensin Receptor-Neprilysin Inhibitor
CRT	Cardiac Resynchronization
ER	Estrogen Receptor
GPER	G protein-coupled estrogen receptor
HFmrEF	Heart Failure with mid-range Ejection Fraction
HFpEF	Heart Failure with preserved Ejection Fraction
HFrEF	Heart Failure with reduced Ejection Fraction
HRQL	Health-Related Quality of Life
ICD	Implantable Cardioverter Defibrillator
LV	Left Ventricle
LVEF	Left Ventricle Ejection Fraction
SGLT2i	Sodium-Glucose Transport Protein 2 inhibitors
